# Retinal toxicity in a multinational inception cohort of patients with systemic lupus on hydroxychloroquine

**DOI:** 10.1136/lupus-2022-000789

**Published:** 2022-11-17

**Authors:** Celline C Almeida-Brasil, John G Hanly, Murray Urowitz, Ann Elaine Clarke, Guillermo Ruiz-Irastorza, Caroline Gordon, Rosalind Ramsey-Goldman, Michelle A Petri, Ellen M Ginzler, Daniel J Wallace, Sang-Cheol Bae, Juanita Romero-Diaz, Mary-Anne Dooley, Christine Peschken, David Isenberg, Anisur Rahman, Susan Manzi, Søren Jacobsen, S Sam Lim, Ronald van Vollenhoven, Ola Nived, Andreas Jönsen, Diane L Kamen, Cynthia Aranow, Jorge Sánchez-Guerrero, Dafna D Gladman, Paul R Fortin, Graciela S Alarcon, Joan T Merrill, Kenneth Kalunian, Manuel Ramos-Casals, Kristjan Steinsson, A Zoma, Anca D Askanase, Munther Khamashta, Ian N Bruce, Murat Inanc, Luck Lukusa, Sasha Bernatsky

**Affiliations:** 1 Centre for Outcomes Research and Evaluation (CORE), Research Institute of the McGill University Health Centre, Montreal, Québec, Canada; 2 Rheumatology, Dalhousie University, Halifax, Nova Scotia, Canada; 3 Schroeder Arthritis Institute, Krembil Research Institute, Center for Prognosis Studies in the Rheumatic Diseases, Toronto Western Hospital, University of Toronto, Toronto, Ontario, Canada; 4 Division of Rheumatology, Cumming School of Medicine, University of Calgary, Calgary, Alberta, Canada; 5 Autoimmune Diseases Research Unit, Biocruces Bizkaia Health Research Institute, Barakaldo, Spain; 6 Rheumatology Research Group, University of Birmingham, Birmingham, UK; 7 Medicine/Rheumatology, Northwestern University, Evanston, Illinois, USA; 8 Division of Rheumatology, Johns Hopkins University School of Medicine, Baltimore, Maryland, USA; 9 Medicine/Rheumatology, SUNY Downstate Medical Center, New York, New York, USA; 10 Cedars-Sinai/David Geffen School of Medicine at UCLA, Cedars-Sinai Medical Center, Los Angeles, California, USA; 11 Rheumatology, Hanyang University Hospital for Rheumatic Diseases, Hanyang University Institute for Rheumatology and Hanyang University Institute of Bioscience and Biotechnology, Seoul, Korea; 12 Immunology and Rheumatology, Instituto Nacional de Ciencias Médicas y Nutrición Salvador Zubirán, Mexico City, Mexico; 13 Medicine, The University of North Carolina at Chapel Hill, Chapel Hill, North Carolina, USA; 14 Rheumatology, University of Manitoba, Winnipeg, Manitoba, Canada; 15 Medicine (Rheumatology), University College London, London, UK; 16 Allegheny Singer Research Institute, Allegheny Health Network, Pittsburgh, Pennsylvania, USA; 17 Copenhagen Lupus and Vasculitis Clinic, Copenhagen University Hospital Center for Rheumatology and Spine Diseases, Copenhagen, Denmark; 18 School of Medicine, Emory University, Atlanta, Georgia, USA; 19 Department of Rheumatology, Amsterdam Rheumatology and Immunology Center, Amsterdam, The Netherlands; 20 Department of Clinical Science, Rheumatology, Lund University Faculty of Medicine, Lund, Sweden; 21 Medicine, Medical University of South Carolina, Charleston, South Carolina, USA; 22 Autoimmune and Musculoskeletal Disease, Feinstein Institute for Medical Research Fertility Research Laboratory, Manhasset, New York, USA; 23 Center for Prognosis Studies in the Rheumatic Diseases, Toronto Western Hospital, Toronto, Ontario, Canada; 24 Medicine-Rheumatology, Universite Laval, Quebec, Quebec, Canada; 25 Medicine, Division of Clinical Immunology and Rheumatology, The University of Alabama at Birmingham, Birmingham, Alabama, USA; 26 Arthritis & Clinical Immunology Program, Oklahoma Medical Research Foundation, Oklahoma City, Oklahoma, USA; 27 Division of Rheumatology, Allergy and Immunology, University of California San Diego, La Jolla, California, USA; 28 Department of Autoimmune Diseases, Hospital Clínic, Universitat de Barcelona, Barcelona, Spain; 29 Rheumatology, Department of Obstetrics and Gynecology, Landspitali University Hospital, Reyjavik, Iceland; 30 Lanarkshire Centre for Rheumatology, Hairmyres Hospital, East Kilbride, UK; 31 Rheumatology, Columbia University Medical Center, New York, New York, USA; 32 Rayne Institute, St Thomas Hospital, London, UK; 33 Arc Epidemiology Unit, The University of Manchester, Manchester, UK; 34 Internal Medicine Rheumatology, Istanbul University, Fatih, Istanbul, Turkey; 35 Division of Rheumatology, McGill University Health Centre, Montreal, Quebec, Canada

**Keywords:** epidemiology, lupus erythematosus, systemic, outcome assessment, health care

## Abstract

**Objective:**

To evaluate hydroxychloroquine (HCQ)-related retinal toxicity in the Systemic Lupus International Collaborating Clinics (SLICC) inception cohort.

**Methods:**

Data were collected at annual study visits between 1999 and 2019. We followed patients with incident SLE from first visit on HCQ (time zero) up to time of retinal toxicity (outcome), death, loss-to-follow-up or end of study. Potential retinal toxicity was identified from SLICC Damage Index scores; cases were confirmed with chart review. Using cumulative HCQ duration as the time axis, we constructed univariate Cox regression models to assess if covariates (ie, HCQ daily dose/kg, sex, race/ethnicity, age at SLE onset, education, body mass index, renal damage, chloroquine use) were associated with HCQ-related retinal toxicity.

**Results:**

We studied 1460 patients (89% female, 52% white). Retinal toxicity was confirmed in 11 patients (incidence 1.0 per 1000 person-years, 0.8% overall). Average cumulative time on HCQ in those with retinal toxicity was 7.4 (SD 3.2) years; the first case was detected 4 years after HCQ initiation. Risk of retinal toxicity was numerically higher in older patients at SLE diagnosis (univariate HR 1.05, 95% CI 1.01 to 1.09).

**Conclusions:**

This is the first assessment of HCQ and retinal disease in incident SLE. We did not see any cases of retinopathy within the first 4 years of HCQ. Cumulative HCQ may be associated with increased risk. Ophthalmology monitoring (and formal assessment of cases of potential toxicity, by a retinal specialist) remains important, especially in patients on HCQ for 10+ years, those needing higher doses and those of older age at SLE diagnosis.

**WHAT IS ALREADY KNOWN ON THIS TOPIC**

- Despite the beneficial effects of hydroxychloroquine (HCQ) in SLE, retinal toxicity is a major concern for many physicians and patients. Evidence supporting current guidelines is derived from a few studies, none of which were based in incident SLE samples.

**WHAT THIS STUDY ADDS**

- Although retinal toxicity is a rare event in incident users of HCQ (overall incidence was 1 in 1000 patient-years), our first case of retinal toxicity occurred at 4 years of HCQ therapy. Unadjusted risk of retinal toxicity was higher in older age patients at SLE diagnosis.
- Overall, cumulative risk was <1% within the first 10 years of continuous HCQ use, but we had relatively few patient-years at 10 years and beyond.

**HOW THIS STUDY MIGHT AFFECT RESEARCH, PRACTICE OR POLICY**

- Most people on HCQ did not develop retinal toxicity within the average of 7.4 years of follow-up.
- Nevertheless, cumulative HCQ duration may be associated with increased risk, and ophthalmology assessment every 6–12 months is important, especially in those on HCQ for 10 years or more, those needing higher doses and those of older age. Patients suspected of retinal toxicity should be assessed formally by a retinal expert with appropriate tests to ensure diagnosis and to avoid unnecessary cessation of HCQ given risk of flare when doses reduced or stopped.
- More work is needed to identify which patients are at highest and lowest risk of retinal toxicity, so that HCQ tapering or withdrawal is offered to the right patients, at the right time, for the right reasons.

## Introduction

Hydroxychloroquine (HCQ) is a mainstay in SLE treatment. However, long-term HCQ use may induce retinal toxicity, a serious event that potentially leads to blindness.^1 2^ Retinal damage is of major concern for physicians and patients since there is no specific treatment (other than discontinuing the drug) and serious vision loss can ensue.^3^

Recommendations of the American Academy of Ophthalmology (AAO) were revised in 2016 so that the current recommended maximum daily dose is 5 mg/kg (based on actual body weight).^1^ Potential risk factors for HCQ/chloroquine (CQ) retinopathy, including high daily dosage, cumulative HCQ and reduced renal function have been studied only in patients with prevalent SLE.^1 2 4–10^ Some have misgivings that AAO guidelines are based on suboptimal evidence.^6^

We aimed to evaluate the temporal relationship between HCQ use and retinal toxicity in an incident SLE cohort. We also investigated demographic and clinical characteristics associated with HCQ-induced retinal toxicity.

## Methods

### Study population and design

The Systemic Lupus International Collaborating Clinics (SLICC) cohort is a multinational inception cohort for the study of SLE outcomes.^11^ From 1999 to 2011, a cohort of patients with newly diagnosed SLE was recruited from 33 SLICC sites in Europe, Asia and North America, as previously described.^12^ Briefly, patients meeting American College of Rheumatology (ACR) classification criteria for SLE^13^ were enrolled within 15 months of diagnosis. Data were collected per protocol at enrolment and annually until 2019.

For this study, we identified all patients from the SLICC cohort on HCQ therapy at any point. Time zero was the study visit where HCQ use was first recorded and patients were followed until the outcome of interest, end of study period (April 2019), death or loss to follow-up.

### Outcome

The outcome of retinal toxicity, assessed in all HCQ-exposed patients. We first identified patients with retinal damage recorded in the SLICC/ACR Damage Index (SDI) item for retinal changes. The SDI (including the item for retinal toxicity) was completed in a standard fashion by the SLICC physician investigators, at the annual study visit of each patient. The SDI is normally completed by the physician investigator using a combination of review of prior notes, patient history and physical examination. For the purposes of our study, for each patient with damage noted on the SDI retinal item, the physician investigator responsible was asked to confirm if the recorded retinal damage was indeed antimalarial-related retinal toxicity (vs other types of retinal damage). This was generally done by the physician investigator by referring to clinical notes; the physician investigator not only generally was the same evaluator from 1 year to the next, but also provided care between visits, so would normally be aware of an outcome like retinal toxicity. We did not record how often patients underwent ophthalmology testing; the assumption was that patients were encouraged to be evaluated at least yearly as per guidelines (although in the real world, patients may miss ophthalmology visits).

### Exposure

At annual follow-up visits, average HCQ daily dose since the last assessment was recorded. Cumulative duration of HCQ was defined as the total time the patient was exposed to HCQ since time zero (first visit on HCQ) until the patient experienced the event (or end of study period, death or loss to follow-up). We also calculated the mean daily HCQ dose for each patient throughout the study. HCQ doses per kilogram were calculated by dividing the average HCQ dose at each annual visit by the patient’s recorded body weight. We then summed the average daily dose of all visits and divided by the total number of visits with HCQ use. In addition, we determined the number of patients who had ever been exposed to doses above the current recommendation of 5 mg/kg/day.^1^

### Covariates

Patients were described in terms of the following characteristics assessed at time zero: age at SLE diagnosis (continuous), sex, race/ethnicity (white, black, Asian or other), high school education or less versus college/university education, geographical location (North America, Europe or Asia), SLE duration (continuous), body mass index (BMI, continuous), current smoking (yes/no), high disease activity (≥4 points on SLE Disease Activity Index 2000^14 15^), presence of renal damage, based on the SDI,^16^ current prednisone (yes/no), current immunosuppressive agents (azathioprine, methotrexate or mycophenolate mofetil) and current biological agents (rituximab or belimumab). We also identified if patients had ever used CQ.

### Analyses

Descriptive statistics included calculation of means with SD for continuous variables and number with proportions for categorical ones. We plotted a Kaplan-Meier curve for probability of retinal toxicity related to cumulative duration of HCQ therapy, stratified by average daily dose in mg/kg.

Using the cumulative HCQ duration as the time axis, we constructed univariate Cox regression models with each covariate to identify potential predictors of HCQ-induced retinal toxicity. Due to the low number of events, we were unable to produce meaningful multivariate models. All analyses were conducted with SAS V.9.4 (SAS Institute).

## Results

A total of 1460 patients (89% female) were included. Mean age at SLE diagnosis was 34.7 (SD 13.3) years; mean SLE duration at time zero (first visit on HCQ) was 1.2 (SD 2.1) years, that is, about 14 months; median SLE duration at time zero was 7 months (IQR 2.8 months–1 year). Patients were followed for an average of 7.4 (SD 4.4) years (median 7.3, range 4.0–17 years). Demographic and clinical characteristics of patients are shown in table 1.

**Table 1:** Baseline characteristics of patients with SLE (N=1460)

Characteristics*	N (%)	N missing (%)
Male sex	162 (11.1)	0
Race		15 (1.0)
White	755 (51.7)	
Black	248 (17.0)	
Asian	233 (16.0)	
Other	209 (14.3)	
No college/university education	539 (36.9)	27 (1.8)
Geographical location		0
North America	907 (62.1)	
Europe	390 (26.7)	
Asia (South Korea)	163 (11.2)	
Body mass index (SD)	24.8 (5.9)	58 (4.0)
Smoker	206 (14.1)	1 (0.1)
SLEDAI-2K ≥4	621 (42.5)	16 (1.1)
Renal damage	73 (5.0)	28 (1.9)
Prednisone	1034 (70.8)	0
Biologics	26 (1.8)	0
Immunosuppressive	697 (47.7)	0
Chloroquine	36 (2.5)	0

*At time zero, unless otherwise indicated.

SLEDAI-2K, SLE Disease Activity Index 2000.

Retinal toxicity was suspected in 26 patients and confirmed in 11 of these (incidence 1.0 per 1000 person-years, 0.8% overall). For the remaining 15 patients in whom retinal toxicity was suspected, retinal changes were confirmed by ophthalmological assessments to be either non-significant changes or changes related to other causes. The average cumulative time on HCQ among those with confirmed retinal toxicity was 7.4 (SD 3.2) years, median and range of 6.3 (4.5–10.8) years. The earliest diagnosis of retinal toxicity occurred after 4 years of HCQ therapy and the lowest average HCQ dose in patients with retinal toxicity was 3.6 mg/kg/day; the highest was 9.3 mg/kg/day. A total of 853 patients (60%) ever used a daily dose of HCQ above 5 mg/kg; as expected, most (86%) of these events occurred prior to AAO guideline publication (July 2016).

Over follow-up, in addition to the 11 patients with retinal toxicity, another 420 patients stopped HCQ during follow-up. Only 68 patients stopped HCQ in the first 5 years of follow-up (including three in the first year of follow-up), and these were about equally distributed between North American centres (N=32, 47.1% of 68) and other jurisdictions (N=36, 52.9% of 68). Table 2 indicates the age, sex, and race/ethnicity of those who stopped or did not stop HCQ at any time during follow-up, suggesting fairly similar baseline demographics in these two groups.

**Table 2:** Characteristics of patients continuing or stopping HCQ during follow-up

	Continued HCQ N=1029	Stopped HCQ N=431
Female sex, N (%)	911 (88.5)	387 (89.8)
White race/ethnicity, N (%)	574 (55.8)	181 (42.0)
Mean age at SLE diagnosis (SD)	35.3 (13.3)	33.4 (13.0)
Centre, N (%)		
Canada	264 (25.7)	97 (22.5)
USA	331 (32.2)	82 (19.0)
Mexico	41 (4.0)	82 (19.0)
UK	196 (19.0)	76 (17.6)
Korea	86 (8.4)	61 (14.2)
Other	111 (10.8)	33 (7.7)

HCQ, hydroxychloroquine.

**Figure 1:**
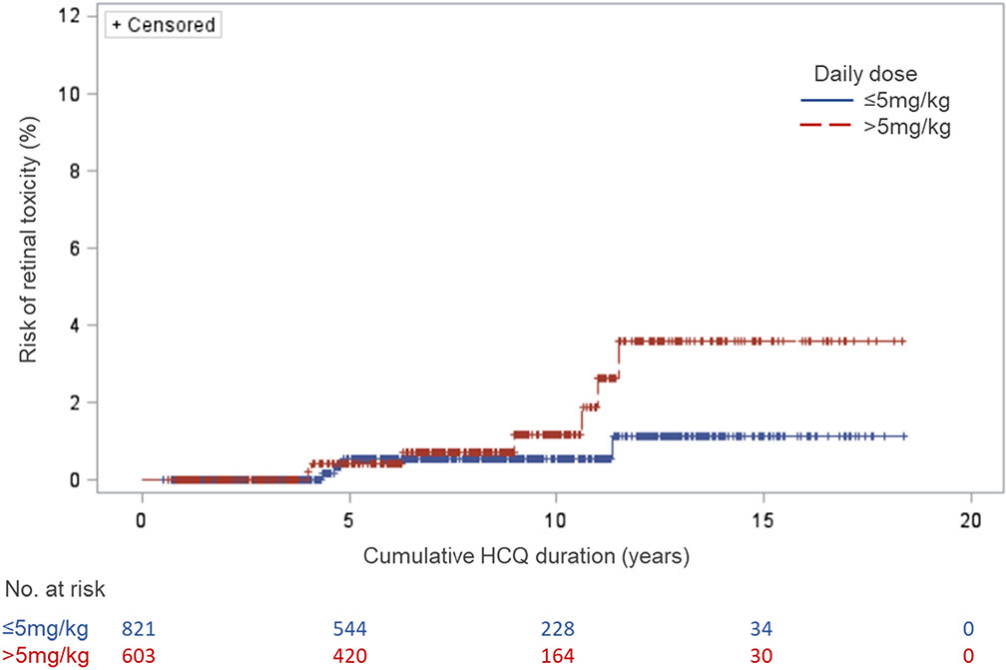
Kaplan-Meier curves for retinal toxicity at two HCQ mean daily dose levels. HCQ, hydroxychloroquine.

In the Kaplan-Meier curves (figure 1), the crude probability of retinal toxicity was less than 1% until around 10 years of cumulative HCQ use. Figure 1 suggests that the risk of retinal toxicity appeared to increase over time, particularly for patients using HCQ doses above the recommendation (>5 mg/kg). However, follow-up beyond 10 years was available in only a handful of patients (31 in the group <5 mg/kg and 30 in the group >5 mg/kg). Thus, we cannot comment on the statistical significance of differences in outcomes for these two groups.

Our hazards regression analyses (table 3) identified that the unadjusted risk of retinal toxicity increased with the age at SLE diagnosis. Non-significant trends for greater risk were observed for those using higher daily HCQ doses, in males and in black patients. None of the patients with retinal toxicity lived in Asia, were currently smoking, had renal damage or had used CQ.

**Table 3:** Univariate Cox regression for retinal toxicity in HCQ-exposed patients with SLE

Characteristics	HR (95% CI)
Mean daily HCQ dose (mg/kg)	1.42 (0.91 to 2.21)
Mean daily HCQ dose >5 mg/kg/day	2.35 (0.69 to 8.04)
Ever used HCQ >5 mg/kg/day	1.25 (0.33 to 4.73)
Male sex	1.93 (0.42 to 8.92)
Race/ethnicity	
White	Reference
Black	1.16 (0.24 to 5.77)
Asian	0.94 (0.19 to 4.65)
Other	0.94 (0.11 to 7.86)
Age at SLE diagnosis in years	1.05 (1.01 to 1.09)
No college/university education at time zero	1.02 (0.30 to 3.49)
Geographical location	
North America	Reference
Europe	0.77 (0.20 to 2.91)
Asia	0
Mean body mass index	1.00 (0.90 to 1.11)
Smoker at time zero	0
SLEDAI-2K ≥4 at time zero	0.81 (0.24 to 2.78)
Renal damage at time zero	0
Ever use of chloroquine	0

HCQ, hydroxychloroquine; SLEDAI-2K, SLE Disease Activity Index 2000.

## Discussion

The incidence of HCQ-induced retinal toxicity in our multinational inception cohort of 1460 patients with SLE was 1.0 per 1000 person-years (0.8% over the study interval). We did not see any cases of retinal toxicity within the first 4 years of HCQ use and most people on HCQ did not develop retinal toxicity (although we had data on less than 70 patients after 15 years of cumulative HCQ use). Trends suggesting more risk in patients after 10 years, especially with daily dose >5.0 mg/kg, are consistent with beliefs that high daily dose and cumulative time on HCQ are risk factors for retinopathy.^5^ The prevalence of retinal damage associated with long-term use of antimalarial drugs reported in previous studies varies widely,^2 8 10 17 18^ likely due to differences in study design and outcome ascertainment. Several studies observed that risk of retinal toxicity increases significantly beyond 7–20 years of HCQ use,^2 8 18^ but prior analyses have never studied an incident patient cohort, as we did. Risk of retinopathy was numerically lower (<1%) in the first 10 years of exposure in our analyses, compared with the risk (4%) after 11–17 years. However, we had more than 10 years of observation for only a minority of subjects; thus, results past 10 years of follow-up have to be viewed with some uncertainty. Moreover, in Figure 2, as person-years of observation increase, there are more than one factor at play in terms of retinopathy—one is duration on HCQ, but another is increasing use over time of sensitive techniques (ie, spectral-domain optical coherence tomography, SD-OCT) to detect retinal pathology. Differentiation of these two effects is difficult. However, the work of Petri *et al* showed 1%–2% risk of retinopathy in patients with SLE during the first 10 years of HCQ therapy, but 11.5% in those who had taken HCQ for 16–20 years.^8^ Our results seem generally consistent with this, although Petri *et al* also found greater retinal toxicity in patients with higher BMI (which we were unable to detect) and HCQ levels (which were unavailable in the patients we studied).

The current AAO (2016)^1^ and European Alliance of Associations for Rheumatology (2019)^19^ recommendations caution against exceeding 5 mg/kg (actual body weight) of HCQ per day. Both guidelines base their recommendations mainly on a single study conducted in the USA in 2014.^2^ This study (which only included patients taking HCQ for >5 years) observed that patients using 4–5 mg/kg HCQ had <2% risk of retinopathy within the first 10 years of use, while patients exceeding 5 mg/kg had approximately a 10% risk in the same period. In our study, patients had less than 1% risk in the first decade of use. After 10 years, we observed a non-significant trend for higher risk, particularly among patients with a mean daily dose >5 mg/kg (but the 95% CIs around our estimates were very wide, table 2).

Recommendations from ophthalmology and rheumatology associations include good practices on screening for retinal toxicity, such as annual ophthalmology examination particularly in patients with >5 years of use and/or other risk factors (dose >5 mg/kg/day, renal impairment and other factors).^5 20 21^ Interestingly, three of our patients with retinal toxicity did not have any of these risk factors.

Our univariate analysis suggests that older age at SLE diagnosis may be associated with higher risk of retinopathy. Age was not included as a major risk factor in the 2016 AAO guidelines,^1 22^ but more recent studies also found older age to be a predictor in unadjusted analyses.^8 10^ The loss of retinal neurons with older age may set the stage for the clinical development of antimalarial retinal toxicity.^23^ However, the effect observed could be due to confounders. Older patients may be more adherent to drugs (higher exposure) and to ophthalmology monitoring (higher chances of event detection). Older patients may also have additional ocular (including retinal) pathology, and in some cases, this could potentially alter the likelihood of being diagnosed (correctly or incorrectly) with retinal toxicity. Given that there is no easily available replacement for HCQ in SLE, it is important to highlight the importance of collaborating closely with ophthalmology in the setting of retinal changes, particularly when there is some doubt as to the aetiology.

It is worth noting that one study has observed that patients with longer duration of antimalarial exposure are also more likely to miss their ophthalmology appointments,^24^ an important reminder that adherence to ophthalmological monitoring may decrease as risk for retinal toxicity is increasing.

Pharmacologically, CQ may have greater potential for retinal toxicity than HCQ. However, CQ use was uncommon, and none of the 36 patients who used CQ developed retinal toxicity during the study period.

Some key potential limitations must be mentioned. Our outcome was captured using the SDI, with cases of retinal damage being verified by the research investigator, to confirm that retinal changes were due to HCQ toxicity. Thus, we would miss cases not detected clinically and not recorded by the clinical investigator in the SDI, particularly when mild subclinical retinal changes are detected by high-sensitivity techniques (ie, SD-OCT) which have become a regular part of routine ophthalmological surveillance for at least a decade. In a recent study of 110 patients (99% receiving HCQ doses <5 mg/kg/day), no clinically significant retinal changes by SD-OCT were found during a 5-year follow-up study.^25^ Of course, even the interpretation of these tests is highly dependent on the expertise of the ophthalmologists. For these reasons, we do not want our study to give prescribing rheumatologists a false sense of security about the ocular risks of HCQ.

We observed only 11 events over the study interval, which limited our power to make strong inferences about most demographic and clinical characteristics in terms of their association with HCQ-induced retinal toxicity. The relatively short follow-up is a potential limitation, and it is imperative that further study of retinal toxicity in incident SLE be undertaken. We did not assess adherence to HCQ, which many studies have shown can be suboptimal in SLE.^26^ However, almost no observational studies of drug effects in SLE (or any other disease for that matter) completely account for non-adherence. These limitations were included in our poster presentation of this work at the ACR annual scientific meeting in 2020.^27^

HCQ seems likely to remain a first-line drug in the management of patients with SLE for the foreseeable future. Ophthalmology screening is important for early detection of retinal toxicity which needs confirming by a retinal specialist. We need more work to prospectively identify which patients are at highest and lowest risk of retinal toxicity. HCQ tapering or withdrawal should only be offered to the right patients and at the right time for the right reasons as this can be associated with risk of flare,^28^ and other drugs may have more risk than continuing HCQ at appropriate dosing if there is no definite evidence of HCQ retinal toxicity.

## Data Availability

All data relevant to the study are included in the article or uploaded as supplemental information.
